# Retrograde vs. antegrade flexible nephroscopy for detection of residual fragments following PNL: a prospective study with computerized tomography control

**DOI:** 10.1590/S1677-5538.IBJU.2018.0695

**Published:** 2019-07-27

**Authors:** Mehmet İlker Gökce, Omer Gülpinar, Arif Ibiş, Muratcan Karaburun, Eralp Kubilay, Evren Süer

**Affiliations:** 1 Department of Urology, Ankara University School of Medicine, Adnan Saygun Caddesi, Altindağ, Ankara, Turkey

**Keywords:** Nephrolithotomy, Percutaneous, Surgical Procedures, Operative, Ureter

## Abstract

**Introduction:**

The main aim of stone surgery is to establish stone free status. Performing flexible nephroscopy is an effective tool in this manner. The aim of this study was to evaluate the role of retrograde flexible nephroscopy for detection of residual fragments following percutaneous nephrolithotomy (PNL) in comparison with antegrade approach.

**Materials and Methods:**

Data of 137 patients underwent ECIRS was collected prospectively. In all cases following stone clearance, collecting system was checked for residual fragments. First antegrade than retrograde flexible nephroscopy was performed and success rates to reach all calices and detection of residual fragments were noted. All patients underwent CT and success rate of antegrade and retrograde approaches were compared. PPV and NPV of retrograde approach to detect residual fragments were calculated.

**Results:**

Antegrade and retrograde nephroscopy successfully accessed all of the calices in 101 (73.7%) and 130 (94.9%) patients respectively (p<0.0001). Residual fragments were observed in 18 (13.1%) patients following antegrade flexible nephroscopy. Retrograde approach identified residual stones in 17 more cases. These cases were treated with flexible nephroscopy or secondary percutaneous tract. Postoperative CT revealed residual stones in 10 (7.3%) patients. PPV and NPV of retrograde flexible nephroscopy were 83.3% and 96.2%.

**Conclusions:**

Flexible nephroscopy effectively detects residual fragments following PNL. Retrograde approach was more successful than antegrade approach to reach all calices. We recommend performing retrograde flexible nephroscopy following PNL especially in complex cases as it has the potential to increase SFR, decrease the need for second look surgery and unnecessary postoperative imaging.

## INTRODUCTION

Percutaneous nephrolithotomy (PNL) is recommended as the gold standard treatment for management of stones > 20 mm in diameter by both EAU and AUA guidelines (1, 2). Also, with miniaturized instruments, stones < 20 mm can also be treated with high success and low complication rates (3). The main aim of stone surgery is to establish the stone free status due to the fact that residual fragments may result in stone related events. In two recent studies employing non-con trast enhanced computerized tomography (NCCT) as the imaging modality postoperatively, stone related events were reported in 31% and 46% of the cases with residual fragments after PNL (4, 5).

Besides prevention of stone related events, detection of residual fragments at the end of PNL is also important for deciding to place a re-entry tube as an exit strategy as well. In a recent study by Harraz et al. the authors evaluated stone free status with fluoroscopy guidance. In this study, surgeon's decision for stone free status has positive and negative predictive values of 92.8% and 72% (6). Portis et al. evaluated the role of surgeon's decision for stone free status with the aid of flexible nephroscopy and reported positive and negative predictive values of 67% and 73% (7). These previous studies proved that fluoroscopic evaluation of the stone free status following PNL is not highly sensitive and specific (6) and additional use of antegrade flexible nephroscopy does not make the evaluation perfect as well (7).

Retrograde intrarenal surgery together with supine PNL was first described by Ibarluzea et al. in 2007 (8) and the term Endoscopy Combined Intrarenal Surgery (ECIRS) was described by Scoffone et al. in 2008 (9). In our experience with ECIRS, performing retrograde flexible nephroscopy is very convenient. We hypothesized that performing the flexible nephroscopy antegradely to evaluate the presence of residual fragments may limit its success for visualizing all of the calices, especially the ones located just next to the calyx entered percutaneously.

To the best of our knowledge current literature lacks studies comparing antegrade and retrograde flexible nephroscopy following PNL. Therefore, the aims of this study were to prospectively evaluate the role of retrograde flexible nephroscopy for detection of residual fragments following PNL in comparison with antegrade flexible nephroscopy and to see if performing a retrograde flexible nephroscopy would dispense the need for postoperative NCCT.

## MATERIALS AND METHODS

In this study, data of 167 consecutive patients underwent ECIRS for renal stones occupying multiple calices at our department between September 2016 and March 2018 were collected prospectively. Thirty patients that did not underwent a postoperative NCCT were excluded from final analysis and data of 137 patients were evaluated. Informed consent was obtained from all individual participants included in the study. All of the operations were performed by a single experienced surgeon in the Galdakao modified supine Valdivia (GMSV) position.

### Operative technique and study design

As the initial step, cystoscopy was performed and a ureteral catheter or a 9.5 / 11.5 Fr ureteral access sheath (Cook, Flexor^®^, Bloomington, IN, USA) was placed in the ureter and retrograde pyelogram was performed by injection of radiopaque contrast material. Renal puncture was performed with aid of fluoroscopy and a hydrophilic guidewire was placed in the collecting system. The MIP-M kit (Karl Storz, Tuttlingen, Germany) was used for mini-PNL cases. One shot dilation with 15 Fr metallic dilator was performed and 16 Fr metallic sheath was placed 12 Fr nephroscope was introduced and laser lithotripsy was performed in all cases. For active stone clearance vacuum cleaner effect was applied. In cases underwent conventional PNL, following guidewire placement, Amplatz renal dilators were used to dilate the tract to 24 Fr diameter and Amplatz sheath was placed 20.8 Fr nephroscope (Richard Wolf, Knittlingen, Germany) was introduced and ballistic lithotripter (Elmed, Ankara, Turkey) was used for stone fragmentation.

In all of the cases, following stone clearance, collecting system was checked for residual fragments with fluoroscopy and presence of any residual fragments was noted. As the second step antegrade flexible nephroscopy was performed. A fiberoptic flexible ureterorenoscope (FLEX-X2?, Karl Storz, Tuttlingen, Germany) was introduced through the metallic sheath and the surgeon attempted to reach all of the calices and position of the flexible ureterorenoscope was checked with fluoroscopy. Success of antegrade nephroscopy to reach all of the calices and detection of residual fragments of any size was noted. As the third step, retrograde flexible nephroscopy was performed again with the same flexible ureterorenoscope. For this purpose, flexible ureterorenoscope was either introduced through the previously placed access sheath or a guidewire was placed through the ureteral catheter and flexible ureterorenoscope was introduced over the guidewire. The surgeon again attempted to reach all of the calices and position of the ureterorenoscope was checked with fluoroscopy. The percutaneous sheath was closed during retrograde ureterorenoscopy to fill the collecting system and expand. Successful access to all of the calices and detection of the residual fragments was again recorded. Any residual fragment was treated at this step either via laser lithotripsy or a nitinol basket (Dakota 1.9 F x 8 mm x 120 cm, Boston Scientific, Boston, MA) was used to extract the stone through the percutaneous sheath. A second percutaneous access was performed in case of unsuccessful treatment of the residual fragment with the flexible nephroscope. Nephrostomy tube was not placed in any of the cases and a 6 Fr JJ stent was placed in all of the cases.

The JJ stent was planned to be extracted 7 to 14 days after surgery and imaging was performed with low dose NCCT prior to JJ stent extraction. All of the NCCT images were evaluated by a single experienced radiologist. Stone free was defined as absence of residual fragments of any size in the postoperative NCCT. The other parameters collected were, age, gender, stone diameter, stone location, stone multiplicity, stone free rate (SFR), and auxiliary procedures.

### Statistical analysis

Statistical analysis was performed with SPSS for Windows, ver. 22.0 (SPSS Inc., Chicago, Illinois, USA). The primary end point of the study was to compare the success rates of antegrade and retrograde ureterorenoscopy for access to all of the calices and detection of residual fragments. The two methods were compared with chi-square test. Also, positive predictive value (PPV), and negative predictive value (NPV) of retrograde flexible nephroscopy for detection of residual fragments were calculated. For statistical significance P value of 0.05 was accepted.

## RESULTS

The mean age of the population was 46.2 ± 5.4 years and 92 (67.2%) of the patients were males. Stones occupying at least 2 more calices in addition to the main stone were present in 103 (75.2%) of the patients and the largest stone was located most commonly in the middle calyx or renal pelvis (50.5%). The demographics, stone related, and operative characteristics of the study population is summarized in Table-1.

**Table 1:** Demographic, stone related, and operative characteristics of the study population.

Parameters		Study population (n=137)
Age, mean ± SD		46.2±5.4
**Gender, n (%)**		
	Male	92 (67.2)
	Female	45 (32.8)
**Stone location, n (%)**		
	Upper calyx	14 (10.2)
	Middle calyx and renal pelvis	64 (46.7)
	Lower calyx	59 (43.1)
Stone size, (mm) mean ± SD		29.6±5.4
Stone density (Hounsfield Unit), mean ± SD		977±118.4
Multiple access n (%)		12 (8.8)
Fluoroscopy time (seconds) mean ± SD		74±15.3
Total surgical time (minutes) mean ± SD		80.6±25.1
Hemoglobin drop (g/dL) mean ± SD		0.8±0.6

Antegrade and retrograde nephroscopy successfully accessed all of the calices in 101 (73.7%) and 130 (94.9%) patients respectively and the difference was statistically significant (p < 0.0001). During the retrograde approach, the reason for failed access to all calices was blurred vision due to bleeding in 5 cases and a narrow caliceal neck in the rest of the 2 cases. All of these inaccessible calices were fluoroscopically free of stones as well. Residual fragments were observed in 18 (13.1%) patients following antegrade flexible nephroscopy. Retrograde approach successfully identified all of these residual stones and additionally residual stones were identified in 17 more cases and therefore residual fragments were detected in 35 (25.5%) of the patients. The residual fragments could not be visualized with fluoroscopy in 10 of the 17 cases detected during retrograde flexible nephroscopy. In 23 of the 35 cases with residual fragments, these fragments were successfully treated with retrograde flexible nephroscopy and a second access was performed in 12 patients. At the end of the procedure, based on retrograde flexible nephroscopy 6 patients were reported to have residual fragments and all of the calices could not be accessed in 7 patients. Therefore, complete stone clearance was reported in 124 (90.5%) patients.

The postoperative NCCT revealed residual stone fragments in 10 (7.3%) patients and 92.7% of the patients were stone free. Five of the patients with a residual fragment were among those 6 patients reported to have a residual fragment at the end of the procedure and one patient possibly passed the residual fragment spontaneously. Three of the other 5 patients were among those 7 patients that retrograde flexible nephroscopy failed to access all of the calices and 2 of the patients were among those 124 patients that were reported to be stone free based on the retrograde flexible nephroscopy at the end of the procedure.

Based on the results of entire study population, the PPV and NPV of retrograde flexible nephroscopy were 83.3% and 96.2%. The results are summarized in Table-2. When we consider the results of the 130 patients whom successful retrograde flexible nephroscopy was performed, the PPV and NPV of retrograde flexible nephroscopy were 83.3% and 98.4%. The results are summarized in Table-3.

**Table 2:** Summary of the stone free rates based on retrograde flexible nephroscopy and NCCT images in the entire population.

		NCCT		
		Residual fragment (+)	Residual fragment (-)	Total
Retrograde flexible nephroscopy	Residual fragment (+)	5	1	6
	Residual fragment (-)	5	126	131
		10	127	137

**Table 3:** Summary of the stone free rates based on retrograde flexible nephroscopy and NCCT images in 130 patients with successful access to all calices.

		NCCT		
		Residual fragment (+)	Residual fragment (-)	Total
Retrograde flexible nephroscopy	Residual fragment (+)	5	1	6
	Residual fragment (-)	2	122	124
		7	123	130

Among the 10 patients with residual fragments, 6 patients had residual fragments less than 4 mm and no further intervention was planned. The diameters of the residual fragments were 4 to 9 mm in rest of the patients and 2 of them underwent shock wave lithotripsy, one patient underwent retrograde intrarenal surgery and one patient underwent ultra-mini PNL.

In total, complications were observed in 8 of the 137 (5.8%) patients. Seven of them were Clavien grade I (fever in 1 patients and hematuria in 6 patients) and one of them was a Clavien grade II complication (bleeding requiring transfusion). Clavien grade III or higher complications were not observed in any of the patients.

## DISCUSSION

The main objective of stone surgery is to establish the stone free status in order to diminish the risk of future stone related events and concomitant surgery. Therefore, detection of the residual fragments at the end of surgery is important and in this study it was found out that performing retrograde flexible nephroscopy is an effective method to search for residual fragments following PNL.

In a recent study, Harraz et al. investigated the value of intraoperative assessment of the surgeon for residual fragments following PNL. The surgeons were asked to report presence of residual fragments and the patients underwent NCCT before hospital discharge. The authors reported that the NPV of the surgeon's assessment for residual fragments was 72%. Also almost 80% of the residual fragments were located in the peripheral calices and surgeons were able to identify residual fragments > 9 mm accurately (6).

The value of performing flexible nephroscopy for detection of residual fragments has been studied by Portis et al. in 2008 (7). In this study, the authors reported that postoperative NCCT detected residual fragments in 9 of 34 (26.5%) patients considered stone free after evaluation with fluoroscopy and flexible nephroscopy. In this study, PNL was performed in prone position and flexible nephroscopy was performed antegrade. The authors reported the NPV of this approach for detection of residual fragments > 4 mm as 100% but the NPV decreased to 73% in case the definition of stone free was absence of any residual fragments (7). The NPV of flexible nephroscopy in this was lower compared to our study (96.2% vs. 73%). A possible explanation for this is performing retrograde flexible nephroscopy increases the rate of successful access to all calices.

In another study, Gücük et al. randomized patients to undergo flexible nephroscopy or not, following PNL. In this study, the authors found out that performing flexible nephroscopy resulted in increased stone free rates in patients with stones of less attenuation values. This is probably associated with failure to notice residual fragments of low attenuation with fluoroscopy. In this study, antegrade flexible nephroscopy was performed and the authors suggested to perform this as a routine procedure especially in case of low density stones (10). However, the authors also mentioned that performing flexible nephroscopy was not convenient due to difficulties in orientation and it requires some expertise. We also observed difficulties of orientation in the collecting system while performing antegrade flexible nephroscopy. However, while doing a retrograde flexible nephroscopy due to the expertise from retrograde intrarenal surgery, it was easier to get oriented in the collecting system.

Another technical difference between the retrograde and antegrade approach is the collapse of the collecting system due to leaking of irrigation fluid through the percutaneous sheath. Gücük et al. also mentioned this in their study that performing flexible nephroscopy with a 16 Fr flexible nephroscope through a 28 Fr Amplatz sheath resulted in blurred vision (10). We also experienced this problem during antegrade flexible nephroscopy and this was probably more prominent in our study due to downwards direction of the sheath in supine position. To overcome this problem, as it is mentioned in the methods section, the percutaneous sheath was closed to allow filing of the collecting system during retrograde flexible nephroscopy.

In the present study, performing retrograde flexible nephroscopy was shown to increase the rate of detection of residual fragments. Therefore, performing retrograde flexible nephroscopy potentially increases the success rates and prevents future stone related events and secondary interventions. Additionally, NPV of retrograde flexible nephroscopy of 98.4% and if the surgeon can access all of the calices with flexible ureterorenoscope, postoperative imaging can be omitted. The disadvantages of performing flexible nephroscopy are prolonged operative times and increased cost. However, with the possible benefit of increased success and decreased re-operation rates, we believe that these disadvantages can be overlooked.

The most important drawback of our study was lack of randomization to undergo antegrade or retrograde flexible nephroscopy and we did not follow the methodology for a prospective comparative study. The comparison of two methods was unfair as observation of residual fragments with antegrade flexible nephroscopy provided hint for retrograde approach to find out residual fragments and therefore, possibly improved the success. However, retrograde approach was more successful for reaching all of the calices as well. Also, all of the patients in the current study were candidates for ECIRS due to multicaliceal stones. This would lead to a selection bias in favor of retrograde flexible nephroscopy and its value is questionable in simple cases such as single renal pelvis stones. Another drawback of the study was evaluation of the residual fragments with NCCT was not performed in postoperative day 1. NCCT was performed 7-14 days postoperatively and therefore, we could not evaluate the immediate SFR and value of retrograde flexible nephroscopy to prevent unnecessary second look surgery.

## CONCLUSIONS

Flexible nephroscopy is a valuable tool during PNL for evaluation and treatment of residual fragments. Retrograde approach was more successful than antegrade approach to reach all of the calices. We recommend performing retrograde flexible nephroscopy following PNL especially in complex cases regardless of the surgeon's impression of stone free status, if the flexible ureterorenoscope is available and patient position is compatible with this approach as it has the potential to increase SFR, decrease the need for second look surgery and unnecessary postoperative imaging.
